# Does a joint development and dissemination of multidisciplinary guidelines improve prescribing behaviour: a pre/post study with concurrent control group and a randomised trial

**DOI:** 10.1186/1472-6963-6-145

**Published:** 2006-11-02

**Authors:** Jody D Martens, Ron AG Winkens, Trudy van der Weijden, Daisy de Bruyn, Johan L Severens

**Affiliations:** 1 Integrated Care Unit, University Hospital Maastricht, The Netherlands; 2 Clinical Epidemiology and Medical Technology Assessment, University Hospital Maastricht, The Netherlands; 3 Department of General Practice, Maastricht University, The Netherlands; 4 Centre for Quality of Care Research (WOK), University of Nijmegen and Maastricht University, The Netherlands; 5 Department of Health Organisation, Policy, and Economics (BEOZ), Maastricht University, The Netherlands

## Abstract

**Background:**

It is difficult to keep control over prescribing behaviour in general practices. The purpose of this study was to assess the effects of a dissemination strategy of multidisciplinary guidelines on the volume of drug prescribing.

**Methods:**

The study included two designs, a quasi-experimental pre/post study with concurrent control group and a random sample of GPs within the intervention group. The intervention area with 53 GPs was compared with a control group of 54 randomly selected GPs in the south and centre of the Netherlands. Additionally, a randomisation was executed in the intervention group to create two arms with 27 GPs who were more intensively involved in the development of the guideline and 26 GPs in the control group.

A multidisciplinary committee developed prescription guidelines. Subsequently these guidelines were disseminated to all GPs in the intervention region. Additional effects were studied in the subgroup trial in which GPs were invited to be more intensively involved in the guideline development procedure. The guidelines contained 14 recommendations on antibiotics, asthma/COPD drugs and cholesterol drugs

The main outcome measures were prescription data of a three-year period (one year before and 2 years after guideline dissemination) and proportion of change according to recommendations.

**Results:**

Significant short-term improvements were seen for one recommendation: mupirocin. Long-term changes were found for cholesterol drug prescriptions. No additional changes were seen for the randomised controlled study in the subgroup. GPs did not take up the invitation for involvement.

**Conclusion:**

Disseminating multidisciplinary guidelines that were developed within a region, has no clear effect on prescribing behaviour even though GPs and specialists were involved more intensively in their development. Apparently, more effort is needed to bring about change.

## Background

Health care expenditure in the Netherlands increases each year, as it does in other European countries. Drug prescribing is an important contributor to this increase in costs, and these costs are expected to continue to increase relevantly in the coming years [1-3]. Medication is not always prescribed effectively: drugs may be prescribed unnecessarily and lower-cost alternatives are not always taken into consideration. The problem can be attributed to demographic factors (growth and ageing of the population), the trend towards new but usually more expensive drugs, patients' increased awareness, pressure from the industry, and last but not least the differences in prescribing practices between primary and secondary care oriented physicians [4,5]. Physicians are also known to prescribe more easily as a result of today's high level of work-related stress and routines [6].

Clinical guidelines may induce small improvements, both in processes and in the outcomes of care [7]. Simple top-down dissemination of mono-disciplinary guidelines alone is not effective [8-12]. To bring about change, a more powerful strategy could be multidisciplinary guidelines and probably even more so if key regional representatives from primary and secondary care are involved in the development. Implementation experts indicate that multistage involvement in the development of a guideline can be a positive contributor to effective implementation of guidelines [13,14]. If national guidelines are involved, adjusting them to the local situation and to specified needs experienced in the target group increases commitment [15,16]. We have therefore adopted an intensive guideline development procedure, involving local representatives of the relevant medical disciplines [17,18].

The intensity is related to the time and energy that was put into the strategy of guideline development to create support among GPs. We set up a study in which we assessed the effects of our intensive multistage guideline development. We hypothesised that this strategy could lead to a modest but relevant change of volumes of prescriptions in the desired direction. As we used two comparisons, we were able to study the effects of dissemination separately from the effects of involving the target group in the preparation and development of the guidelines.

## Methods

### Design and population

**Figure 1:**
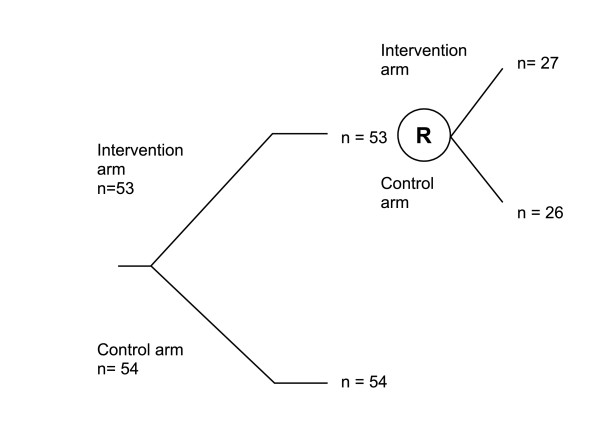
Flowchart of research population and randomised trial in subgroup.

A quasi-experimental study (a pre/post study with a concurrent control group) was executed (fig. 1). Since we had an enormous amount of data from the insurance companies at our disposal (about 700 GPs in the centre and south of the Netherlands), it was practically impossible to include them all. The Maastricht region (intervention region, n = 53 GPs) was compared with a random sample of 54 GPs drawn from the remaining GPs (control group). First, however, an randomised controlled trial (RCT) was carried out within the intervention group; 27 GPs were invited for a more intense role in the development procedure and were compared with 26 GPs in a corresponding control group. The inclusion criteria were: completeness of the GPs' data (no missing data per GP for more than one year) and at least 500 patients in the GPs' practice. As the two groups in the RCT turned out to be comparable, we executed the second comparison: we compared the entire Maastricht region with an external control group, selected at random from all the available data. A pre trial power-calculation was not performed; the study concerned a pragmatic trial, where evaluation was planned alongside an intervention being executed anyway. For this study, only GPs who were not already participating in another regional intervention of similar character, were sent the guideline by post and invited to give comments (n = 53 GPs). The GPs in both designs were not aware of the fact that they were in an evaluation study, because only anonymous volume data were collected from an existing database. There was therefore no need for GPs or patients to give informed consent. The asthma/COPD and cholesterol guidelines were disseminated in March 2002. Baseline data were gathered one year before, and follow-up measurements were made one and two years after the dissemination of each guideline.

### Strategy

The guideline topics were chosen by the steering committee, existing of decision-making representatives (pharmacists, GPs, hospital staff and regional insurance company). Clinical importance in primary care (common problems) and expected health gains were the main criteria in prioritisation. The first multidisciplinary guideline was developed for antibiotics, because non-rational attitudes towards prescriptions and an increase of resistance in micro-organisms have been observed. Over 50% of the antibiotics prescriptions for respiratory tract problems are not used appropriately [19,20]. Guidelines for asthma, COPD and cholesterol have also been developed, due to the high prevalence of these diseases in the population and the corresponding high costs [21]. The guidelines were actually developed by independent multidisciplinary expert teams that included community pharmacists, specialists, GPs and a hospital pharmacist, and were based on Dutch national guidelines, practical experience and consensus. The participating GPs were not involved in the trial.

After the development, we used different methods to validate the conceptual guidelines. For the guideline on antibiotics we started with visiting PTAMs in the region to get comments on the conceptual guidelines and create involvement. For the guidelines on asthma, COPD and cholesterol, the conceptual guidelines were sent by post to a random sample of GPs within the intervention group, and those GPs were asked to comment and encouraged to do so. The guideline committee stressed the importance of knowing the GPs' comments, and that the comments would be taken very seriously in finalising the guideline. Due to the low response rate and based on the expertise of the expert teams, a number of key individual GPs were also contacted in a more active way to generate comments. The guidelines were finalised by presenting the GPs comments to the multidisciplinary expert teams. The Integrated Care Unit of the University Hospital of Maastricht provided both the expertise and the facilitating conditions to disseminate the guidelines within the region.

All the GPs in the intervention group received the finalised paper guidelines in a ring binder by post. The antibiotics guideline was sent out in 2001 and the asthma, COPD and cholesterol guidelines were forwarded in 2002.

### Data collection and analysis

Data were supplied by the two largest insurance companies in the region, which implied that about 70 percent of the total population in the region was covered.

Prescription data were gathered retrospectively per GP per month during the period 2001–2004. Expected directions of change have been defined based on the detailed recommendations contained in the guidelines, in combination with estimates based on the expertise of the initially involved key regional representatives. The drugs that were selected and the desired directions of change are listed in tables 1 and 2, in the first and second columns.

The effect of the strategy and the differences in the pre/post changes between groups were tested with unpaired Student's t-tests and Mann Whitney tests using SPSS 12.0.1 software. Short-term effects were calculated one year after the dissemination of the guidelines, and long-term effects two years after. We set our alpha conservatively at 0.01 because of the multiple testing.

## Results

As far as we can ascertain, the GPs in the two groups did not differ on the main characteristics. The mean age of male GPs in the intervention group was 50.0 years and 53.3 in the control group. The mean age of the female GPs in the intervention group was 48.8 years and 45.5 in the control group. Overall, the percentage of male GPs was only slightly different in both groups: in the intervention group, 85%(45) of the GPs were male, as compared with 76%(41) in the control group. Table 1 presents the mean total number of drug prescriptions per GP per year, standardised per 1000 enlisted patients over a baseline measurement (2001 or 2002), the short-term effects (one year) and long-term effects (two years). Table 1 also presents the absolute changes in the short term and long term per 1000 enlisted patients per GP. Table 2 presents the same data for the random sample of GPs who were invited for a more intense role in the development procedure.

Less than 10% of the GPs that were invited to play a greater role actually commented on the conceptual guidelines. The exact number varied according to the specific guideline. Most of the comments did not relate to content, but rather concerned questions about the development procedure.

For the guideline dissemination (quasi experiment), results show that a significant change in the desired direction was found for one specific antibiotic (mupirocin): a reduction of one prescription per 1000 patients compared to an increase of one (p = 0.0014) in the short term. No significant effect was seen for other antibiotics, either in the short term or the long term. For cholesterol, significant long-term effects were found; an increase of 112 prescriptions per 1000 patients in the intervention group compared to an increase of 54 in the control group (p < 0.0001). No significant effect was seen in the short term or long term for any asthma and COPD drugs.

For antibiotics and cholesterol, no additional effects were found in the randomised study of GPs with a greater involvement (table 2). For asthma and COPD drugs, significant changes were found in the control group instead of the intervention group. Instead of the desired increase, we found a decrease of 9 prescriptions per 1000 patients in the intervention group compared with a decrease of one prescription in the control group for inhaled corticosteroids for asthma (p = 0.0099).

## Discussion

We found no clear impact of disseminating the rigorously developed multidisciplinary guidelines on the prescribing performance of GPs. A significant effect was found for two drugs: mupirocin and cholesterol lowering drugs. The change in mupirocin however does not seem very relevant, because it is rarely prescribed and by and large prescribed for minor ailments. The long-term effect for cholesterol lowering drugs is interesting. The standard deviation of prescribing drugs per GP turned out to be unexpectedly large. Although no significant effects were seen for the other drugs, only small changes can be seen on other indicators. The invitation for GPs to be involved in the development procedure caused no desirable effects in the subgroup.

There are some issues for consideration. First, although unlikely the quasi-experimental design cannot guarantee that the observed changes are the result of the strategy. It is possible that the effects found were caused by coincidence or by an unknown non-specific factor. Although the intervention group in both designs seems to be higher in volumes of prescription at baseline, both groups seem comparable in the exposure to quality improvement strategies that have been performed otherwise in the region. A second issue for consideration is the pragmatic base of the trial in which two regions were compared. This has caused a restriction in the number of GPs in the trial. We were not in the position to improve this. Together with the relatively high variation among the primary variable (volumes of prescription per GP per 1000 patients per year) the power of the study was negatively influenced. Another power calculation executed after ending the study, based on the large standard deviations and the number of GPs, showed that this resulted in a power of 40%. In this study only large differences could be detected. We found a few significant results, although the significant effect of mupirocin is not so interesting because mupirocin is rarely prescribed and the disease is a minor ailment. Given the fact that this study was no selected sample and the participating GPs were a fine representation for the Dutch GPs other results are not expected in larger trials. Besides, when evaluating this intervention in an economic study we wonder if the changes in prescribing behaviour are in balance to such huge efforts made in investment and implementation costs.

Only volume data of prescriptions were used in this initial stage of the study. These figures are therefore not linked to any diagnosis. However, some drugs are prescribed for several different medical problems, while the guideline may contain opposing prescription recommendations. For example, amoxicillin is recommended for the treatment of bronchitis and otitis media acuta, whereas amoxicillin is discouraged in cases of acute cystitis. On the one hand, for statin use, there is a situation of overuse for patients with low risk on cardiovascular disease; on the other hand, there is a situation of underuse for high-risk patients like diabetes patients [21]. It is therefore possible that the results are somewhat underestimated in the sense that possible results were kept hidden. Additional information about diagnosis and cholesterol rates would be needed to make a more precise analysis.

A strong aspect of our study is that long-term effects were measured. For statin-prescription, the initial non-significant effect appeared to be significant at the long run. A positive factor that may have contributed to an increased willingness to follow the cholesterol medication guideline is that our strategy concerns guidelines that have been formulated by representatives from all disciplines and was intended to improve prescription behaviour in both primary care physicians ánd medical specialists.

Overall, the implementation strategy only generated limited results. Implementation experts indicate that if guidelines are developed by the clinicians who are to use them this will be a positive contributor to the effect of guidelines. Achieving consensus on draft guidelines and creating a sense of 'ownership' through a process of development on several levels (central, local and individual) is essential for successful implementation of guidelines [22,23]. We have paid specific attention to this aspect, firstly by involving representatives from all disciplines in the development of the guidelines. The term intensity is therefore related to the time and energy that was put into the guideline development strategy to create support among GPs. Secondly, we also focused specifically on the doctors' acceptance of the guidelines by including them in the validation procedure. It is not clear why the GPs did not respond to the invitation for comment. Possibly, we were successful in choosing opinion leaders to such extent that GPs did not see the need to comment on guidelines that were prepared by GPs they trusted. A second explanation lies in the assumption that the invitation by mail did not work: only a few GPs responded to the invitation to comment on the guideline. GPs might have had comments but did not think this would have any impact in the final guideline. In short, we did not succeed in getting GPs involved locally in the validation procedure. Apparently this way of getting GPs involved is not appropriate and possibly explains why the effect was only limited.

To create involvement in the validation procedure we searched for a successful method to achieve this. During the validation of the antibiotics guideline we started with visiting PTAMs in the region to get comments on the conceptual guidelines and create involvement. This turned out to be very time consuming and caused much delay in the dissemination of the final guideline. Therefore, during the validation of the asthma and the COPD guideline we chose for a less labour-intensive method by asking GPs to comment by mail. After finishing the analyses for this study, we subsequently started (not included in the data of the manuscript) with inviting GPs to feedback meetings. The results of this method were more convincing. We highly recommend the feedback meetings to be an effective method to create involvement among GPs during the validation procedure.

To obtain a more solid or extensive effect, an additional implementation strategy is desired. Feedback and reminders could be an necessary strategy to change GPs behaviour [24-26]. For example, a computerised decision support system could give feedback to a doctor as soon as the prescription behaviour differs from the behaviour recommended in the guideline. As long as the doctor does not deviate from the guideline, the system will not interfere with the primary care physicians' information system, and the doctor giving the prescription will not be interrupted by the feedback system.

We did not perform an economic evaluation by relating the (change in) costs of prescription to the costs of the interventions (guideline development and dissemination) [27,28]. If we were to choose to compare the total expenses of the intensive development procedure of all guidelines with the cost savings, we would expect all the findings to be negative, because the strategy was time and energy consuming and its impact was only moderate. Before aiming to intensify the development procedure of guidelines, the costs and savings of such a strategy need to be investigated thoroughly and executed when the proceeds of the intervention outweighs the costs of it [29,30].

## Conclusion

Disseminating multidisciplinary guidelines that were developed within a region, has no clear effect on prescribing behaviour even though GPs and specialists were involved more intensively in their development. Apparently, more effort is needed to bring about change.

## Competing interests

The author(s) declare that they have no competing interests.

## Authors' contributions

JM participated in the design and execution of the study, performed the statistical analysis, did the preparation and writing of the manuscript. RW did the concept development and coordination of the study and critical review of the manuscript. TW participated in the data analysis and writing of the manuscript. DB did the development of the databases, performed data-analyses and did critical review of the manuscript. JS did the coordination of the study, drafted the manuscript and did the critical reviewing of the manuscript. All authors read and approved the final manuscript.

## Pre-publication history

The pre-publication history for this paper can be accessed here:



## Figures and Tables

**Table 1:** Mean total number of drug prescriptions per GP per year, standardised per 1000 enlisted patients for the total intervention region versus a matched control group

	**Desired direction of drug**	**pre**		**Short term**		**Δ short term**		**Long term**		**Δ Long term**	
		**I (n = 53)**	**C (n = 54)**	**I (n = 53)**	**C (n = 54)**	**I (n = 53)**	**C (n = 54)**	**I (n = 53)**	**C (n = 54)**	**I (n = 53)**	**C (n = 54)**

**Drug indicators**											
**Antibiotics**^**2**^	↓	639 (551,833)	491 (388,595)	667 (532,812)	489 (386,601)	3 (-56,70)	1 (-30,40)	652 (512,767)	486 (405,602)	-20 (-91,65)	-6 (-40,43)
Chinolones^2^	↓↓	37 (22,53)	24 (13,34)	36 (23,48)	22 (12,33)	1 (-12,9)	1 (-6,5)	34(27,44)	21 (14,31)	-4 (-10,4)	-1 (-7,5)
Nitrofurantoin^1^	↑	26 (19,33)	33 (27,39)	27 (22,33)	33 (26,40)	1 (-4,6)	0 (-3,3)	31(25,37)	37 (30,43)	5 (-1,10)	4 (0,8)
Trimethoprim^1^	↑	27 (20,33)	24 (18,30)	29 (21,36)	24 (18,29)	2 (-2,6)	-1 (-3,2)	26(20,32)	22 (17,28)	-1 (-6,5)	-2 (-6,2)
Amoxicillin with clav.acid^2^	↓	31 (24,51)	27 (18,41)	31 (19,46)	26 (17,41)	-3 (-10,8)	-1 (-8,5)	28(18,45)	28 (19,37)	-2 (-16,11)	0 (-10,5)
Amoxicillin^2^	↓	76 (48,126)	62 (37,94)	69 (34,112)	67 (44,100)	-6 (-53,20)	-1 (-12,8)	54(38,104)	58 (37,109)	-14 (-35,2)	-2 (-17,13)
Doxycyclin^1^	↓	133 (109,156)	83 (71,95)	126 (104,148)	79 (68,90)	-7 (-20,6)	-4 (-10,2)	114(93,135)	73 (62,84)	-19 (-35, -3)	-10 (-16, -4)
Mupirocin^2^	↓	5 (2,20)	5 (1,10)	5 (2,13)	5 (1,16)	-1 (-6,1)	1 (-2,5)*	5(2,11)	3 (1,12)	0 (-6,2)	0 (-3,2)
**Cholesterol synthesis inhibitors (Statins)**^1^	↑↑	182 (154,209)	140 (115,164)	219 (188,249)	162 (136,189)	37 (28,46)	22 (16,29)	294 (248,339)	194 (164,224)	112 (87,137)	54 (41,66)*
**Asthma & COPD**											
Long-term beta2-sympaticomimetics^1^	↑	40 (33,46)	40 (32,47)	37 (31,43)	37 (30,44)	-3 (-6, -1)	-3 (-6,0)	36(29,42)	31 (25,38)	-4 (-8,0)	-8 (-13, -4)
**Asthma**											
Short-term beta2-sympaticomimetics^2^	↓	28 (21,44)	29 (19,35)	29 (18,39)	28 (19,41)	0 (-6,6)	2 (-3,7)	28(19,36)	27 (20,38)	-4 (-8,1)	-1 (-8,5)
Inhaled corticosteroids^2^	↑	21 (14,33)	19 (13,28)	19 (10,28)	21 (13,33)	-3 (-8,0)	-1 (-5,4)	14(9,23)	18 (11,26)	-7 (-11, -2)	-2 (-10,1)
**COPD**											
Short-term beta2-sympaticomimetics^1^	-	62 (54,70)	42 (34,49)	61 (52,70)	42 (34,49)	-2 (-6,3)	0 (-3,4)	59(50,69)	36(29,42)	-3(-8,2)	-6 (-10, -2)
Inhaled corticosteroids^1^	↓	55 (46,64)	51 (42,59)	51 (42,60)	48 (39,57)	-5 (-8, -1)	-3 (-6,1)	41(33,48)	40(32,48)	-15(-20, -10)	-10 (-15, -6)

1. Mean (CI) for normally distributed variables

2. Median (P_25_-P_75 _intervals) for skewed variables

* T-test unpaired/Mann-Whitney p < 0.01

**Table 2:** Mean total number of drug prescriptions per GP per year, standardised per 1000 enlisted patients for the randomised more intensively involved group in the intervention region versus a matched control group

	**Desired direction of drug**	**pre**		**Short term**		**Δ short term**		**Long term**		**Δ Long term**		
		**I (n = 27)**	**C (n = 26)**	**I (n = 27)**	**C (n = 23)**	**I (n = 27)**	**C (n = 26)**	**I (n = 27)**	**C (n = 26)**	**I (n = 27)**	**C (n = 26)**

**Drug indicators**											
**Antibiotics**^**2**^	↓	611 (536,833)	530 (421,665)	643 (413,812)	524 (443,622)	6 (-43,82)	3 (-48,38)	661 (435–734)	526 (418,649)	11 (-92,73)	-7 (-61,47)
Chinolones^2^	↓↓	34 (18,51)	25 (14,36)	27 (12,47)	24 (12,32)	3 (-12,8)	-2 (-8,2)	30 (21,38)	19 (17,33)	-3 (-11,3)	-1 (-7,5)
Nitrofurantoin^1^	↑	23 (15,31)	31 (21,40)	27 (20,35)	31 (21,42)	4 (-3,12)	1 (-3,5)	32 (24,41)	36 (26,46)	9 (1,18)	6 (0,11)
Trimethoprim^1^	↑	32 (22,41)	29 (19,38)	32 (20,44)	28 (20,37)	1 (-6,7)	0 (-4,3)	26 (18,35)	23 (16,31)	-5 (-13,3)	-5 (-11,0)
Amoxicillin with clav.acid^2^	↓	33 (22,68)	28 (16,43)	41 (16,67)	26 (17,45)	5 (-9,19)	-2 (-8,5)	23 (16,64)	30 (21,37)	-3 (-16,10)	3 (-8,10)
Amoxicillin^1^	↓	92 (59,125)	104 (63,145)	57 (37,76)	98 (62,135)	-36 (-75,4)	-5 (-16,6)	78 (37,119)	96 (65,128)	-14 (-36,8)	-7 (-25,10)
Doxycyclin^1^	↓	125 (89,160)	89 (73,105)	113 (82,144)	86 (71,100)	-12 (-37,14)	-4 (-12,5)	103 (74,132)	81 (69,93)	-22 (-52,8)	-8 (-16,0)
Mupirocin^1^	↓	12 (5,20)	7 (4,9)	10 (3,16)	6 (3,9)	-3 (-5,0)	-1 (-3,2)	8 (4,12)	6 (3,8)	-5 (-9,0)	-1 (-3,1)
**Cholesterol synthesis inhibitors (Statins)**^1^	↑↑	150 (119,182)	139 (109,170)	182 (144,220)	166 (129,203)	32 (20,43)	27 (17,36)	229 (185,273)	194 (153,235)	79 (60,98)	55 (35,75)
**Asthma & COPD**											
Long-term beta2-sympaticomimetics^1^	↑	35 (27,44)	36 (28,43)	34 (25,42)	33 (25,41)	-2 (-5,2)	-2 (-7,2)	32 (23,42)	28 (21,35)	-3 (-8,3)	-8 (-14, -2)
**Asthma**											
Short-term beta2-sympaticomimetics^1^	↓	37 (26,48)	35 (23,47)	37 (26,48)	37 (25,49)	0 (-4,4)	2 (-1,6)	34 (24,44)	36 (24,48)	-3 (-8,2)	1 (-4,6)
Inhaled corticosteroids^2^	↑	24 (16,35)	24 (13,28)	22 (12,32)	21 (12,33)	-5 (-8,0)	-1 (-5,5)	15 (10,29)	19 (13,28)	-9 (-11, -4)	1 (-7,1)*
**COPD**											
Short-term beta2-sympaticomimetics^1^	-	65 (51,79)	38 (29,47)	63 (48,79)	39 (29,48)	-2 (-9,6)	1 (-4,5)	61 (45,77)	32 (25,40)	-4 (-12,5)	-5 (-12,1)
Inhaled corticosteroids^1^	↓	59 (45,74)	48 (37,59)	55 (40,70)	46 (34,60)	-5 (-10,1)	-2 (-7,3)	41 (29,53)	39 (28,50)	-18 (-26, -11)	-9 (-16, -2)

1. Mean (CI) for normally distributed variables

2 Median (P_25_-P_75 _intervals) for skewed variables

* T-test unpaired/Mann-Whitney p < 0.01
